# Excess Mortality From Suicide During the Early COVID-19 Pandemic Period in Japan: A Time-Series Modeling Before the Pandemic

**DOI:** 10.2188/jea.JE20200443

**Published:** 2021-02-05

**Authors:** Tatsuhiko Anzai, Keisuke Fukui, Tsubasa Ito, Yuri Ito, Kunihiko Takahashi

**Affiliations:** 1 Department of Biostatistics, M&D Data Science Center, Tokyo Medical and Dental University, Tokyo, Japan; 2 Graduate School of Advanced Science and Engineering, Hiroshima University, Hiroshima, Japan; 3 Department of Medical Statistics, Research & Development Center, Osaka Medical College, Osaka, Japan

**Keywords:** excess mortality, suicide, early COVID-19 pandemic, time series modeling

## Abstract

**Background:**

Suicide amidst the coronavirus disease (COVID-19) pandemic is an important issue. In Japan, the number of suicides in April 2020 decreased by nearly 20% from that in 2019. To assess the impact of an infectious disease pandemic, excess mortality is often discussed. Our main purpose was evaluating excess mortality from suicide in Japan during the early pandemic period.

**Methods:**

We used data on suicides collected by the National Police Agency of Japan until June 2020. We estimated excess mortality during the early pandemic period (March–June 2020) using a time-series model of the number of suicides before the pandemic. A quasi-Poisson model was employed for the estimation. We evaluated excess mortalities by the categories of age and sex, and by prefecture.

**Results:**

No significant excess mortality was observed throughout the early pandemic; instead, a downward trend in the number of suicides for both sexes was noted. For males, negative values of excess mortalities below the lower bound of the 95% prediction interval were observed in April and May. All numbers of females during the period were included in the interval, and the excess mortalities in June were positive and higher than those in April and May. In Tokyo, the number of suicides was below the lower bound throughout the period.

**Conclusion:**

Our results suggest that various changes, such as communication, and social conditions amid the early COVID-19 pandemic induced a decrease in suicides in Japan. However, continuous monitoring is needed to evaluate the long-term effects of the pandemic on suicides.

## INTRODUCTION

During the highly contagious coronavirus disease (COVID-19) outbreak in China that spread across countries worldwide in early 2020, the risk of suicide was noted to be exacerbated.^1^ The COVID-19 pandemic has introduced a plethora of intense new stressors, such as working from home, temporary unemployment, and home-schooling of children, which might produce anxiety, depression, and sleep disturbances.^2^^,^^3^ This is suspected to be related to suicide in people globally.^4^ However, the statistics of the National Police Agency (NPA) in Japan showed that suicides in April fell by nearly 20% from the previous year.^5^

The number of suicides in Japan had fallen continuously for 10 years and dipped to an all-time low of 20,169 in 2019. However, it was the leading cause of death among people aged 10 to 39 in 2018.^6^ Unlike other countries, Japan is not only characterized by suicide among the elderly but also by a high incidence of suicide among middle-aged men.^7^ The variety of factors that affect suicide differ in terms of influence depending on age and sex.^8^^–^^10^ Although the NPA in Japan has summarized the number of suicides, as well as potential reasons for suicide based on suicide notes and other sources, the indirect influence of the COVID-19 pandemic on suicide might not be documented. Therefore, assessing the suicide mortality burden associated with COVID-19 remains challenging.

Excess mortality is a term used in epidemiology and public health that refers to the number of mortalities above and beyond what would be expected under “normal” conditions.^11^^,^^12^ Excess mortality or excess death is typically defined as the difference between the number of mortalities in a specific time period and expected number of mortalities in the same time period.^11^ To assess the impact of infectious diseases, such as the COVID-19 pandemic, some studies evaluated whether the observed number of mortalities deviates from the 95% prediction interval (PI) of the number.^11^^,^^13^ Although excess mortality is a starting point for scientists to assess the overall impact of the pandemic, excess mortality from suicide has not been assessed in Japan.

In this study, therefore, we analyzed the trend of suicides in Japan during the early COVID-19 pandemic period (March–June 2020). We constructed a time-series model based on the number of suicides before the pandemic, considering year and month trends and unemployment rate to represent social conditions. Thereafter, we evaluated excess mortality from suicide compared with the model by sex during the pandemic. We also investigated excess mortality for each sex–age category and for each prefecture. These analyses could reveal the impact of the pandemic on suicide.

## METHODS

### Data and materials

We analyzed data on suicide mortalities per month collected by the NPA of Japan from January 2013 to June 2020. The data were downloaded from the official website of Ministry of Health, Labour and Welfare.^5^ The analysis used the monthly total number of suicides and that for each sex–age category. Age of suicide was categorized into <20, 20–29, 30–39, 40–49, 50–59, 60–69, 70–79, ≥80 years old, and unknown. Provisional estimates of population^14^ by month in Japan for each sex–age category and unemployment rate^15^ for each month were downloaded from the Statistics Bureau of Japan. The monthly population by prefecture was calculated by allocating a monthly population using the composition ratio of each prefecture by year.

Since we exclusively used publicly available aggregate data in this study, formal ethical review was not required.

### Statistical analysis

The number of suicides at time (month) *t*, *y_t_* (*t* = 1,2,…*T*) was given by the following model:

$$y_{t}∼\text{Poisson}(\mu_{t})$$

$$\begin{array}{rl} \log(\mu_{t}) & =\beta_{0}+\beta_{Y}{\text{year}}_{t}+\sum_{M=1}^{12}\beta_{M}I({\text{month}}_{t}=M)\\ & \;+\beta_{U1}u_{t}+\beta_{U2}u_{t-1}+\text{offset}(\log({\text{pop}}_{t})) \end{array}$$

where *year_t_* is a continuous variable as the year at time *t*, *month_t_* is the month at time *t*, *u_t_* and *u_t_*_−1_ are the unemployment rates at time *t* and *t* − 1, and *pop_t_* is the population at time *t*. The parameters *β*_0_, *β_Y_*, *β_M_* (*M* = 1,2,…,12), *β_U_*_1_, and *β_U_*_2_ are coefficients to be estimated, where the monthly effect *β_M_* at time *t* is set to 0 for *M* = 1, and *I*(·) is the indicator function. We applied the quasi-likelihood method for inference considering the overdispersion, assuming that the variance is *Var*(*y_t_*) = *ψμ_t_*. This approach has been used in several studies assessing excess mortality.^11^^–^^13^ An underdispersion was not considered and the classical Poisson model was applied to *y_t_* when *ψ* was less than 1. We fitted the model to *y_t_* by sex. Coefficients were estimated using data for the non-pandemic period from January 2013 to February 2020. We also fitted the model to the number of suicides for each sex–age category and for each prefecture. Data of unknown age category were excluded from the analysis in each sex–age category.

The predicted values of *y_t_*, ${\tilde{y}}_{t}$, for March, April, May, and June 2020 as the pandemic period were derived from the estimated model. To obtain the 95% PI of *y_t_*, we applied a simultaneous procedure based on Bayesian inference discussed in Gelman and Hill.^16^ Excess mortality from suicide was defined as $y_{t}-{\tilde{y}}_{t}$, and the relative difference was defined as $(y_{t}-{\tilde{y}}_{t})/{\tilde{y}}_{t}$. All statistical analyses were performed using R software, version 4.0.2 (R Foundation for Statistical Computing, Vienna, Austria).^17^

## RESULTS

**Figure 1.**
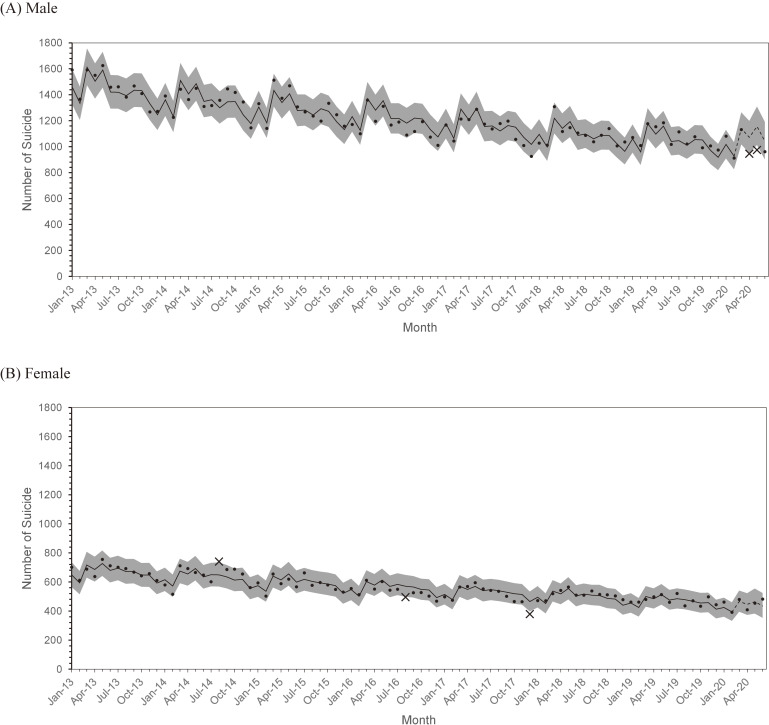
Monthly number of suicides and 95% prediction interval by sex, (A) male and (B) female. Dots: observed number of suicides within the 95% prediction interval; ×: observed number of suicides outside the 95% prediction interval; Solid line: predicted number of suicides in non-pandemic periods; Dashed line: predicted number of suicides in pandemic periods; Gray strip: 95% prediction interval for the number of suicides based on the simulated distribution.

The number of suicides declined from 2013 for both males and females (Figure 1A and Figure 1B). The 95% PI of our model included most observed values during the non-pandemic period; only August 2014, August 2016, and December 2017 for females were outside of the intervals. Most observations for each sex–age category were also included in the PIs (eFigure 1 and eFigure 2).

Table 1 shows excess mortality and relative differences during the pandemic period. For the total number of males, negative values of excess mortality were observed throughout the period. In April and May, the number of suicides was below the lower bound of the 95% PI. Here, we focused on the results for each age category in males. The highest excess mortality was 16.7 in March for the 40–49-year-old age group. The highest relative difference was 15.0% in April for persons aged ≥80 years. However, no observed number exceeded the upper bound of the 95% PI. The observed numbers for the 30–39- and 40–49-year-old age groups in April, and those for 50–59- and 60–69-year-old groups in May were below the lower bound of the interval.

**Table 1.** Number of suicides, excess mortality, and relative difference by sex-age categories

Age group ​ Month	Male					Female				
	
	Population (×10,000)	Observed number	Prediction number (95% PI)^a^	Excess mortality	Relative difference (%)	Population (×10,000)	Observed number	Prediction number (95% PI)^a^	Excess mortality	Relative difference (%)
Total										
March	6,131	1,133	1,138.7 (1019, 1265)	−5.7	−0.5	6,464	480	465.1 (396, 541)	14.9	3.2
April	6,131	946^b^	1,072.4 (951, 1199)	−126.4	−11.8	6,465	410	445.9 (374, 523)	−35.9	−8.0
May	6,128	976^b^	1,155.2 (1014, 1309)	−179.2	−15.5	6,462	455	464.3 (383, 555)	−9.3	−2.0
June	6,129	962	1,041.6 (903, 1192)	−79.6	−7.6	6,464	483	433.9 (353, 524)	49.1	11.3
Under 20 years old										
March	1,071	26	26.2 (16, 39)	−0.2	−0.6	1,019	22	16.7 (8, 29)	5.3	31.6
April	1,071	27	37.4 (23, 54)	−10.4	−27.9	1,018	15	17.3 (7, 31)	−2.3	−13.2
May	1,069	32	31.4 (18, 49)	0.6	1.9	1,018	13	17.0 (7, 33)	−4.0	−23.7
June	1,067	36	32.3 (18, 52)	3.7	11.5	1,016	26	13.4 (4, 28)	12.6	94.3
20–29 years old										
March	652	127	126.1 (98, 157)	0.9	0.7	610	53	52.7 (34, 75)	0.3	0.6
April	654	115	113.9 (87, 145)	1.1	0.9	612	37	49.7 (31, 73)	−12.7	−25.6
May	654	112	116.5 (86, 153)	−4.5	−3.8	613	45	45.5 (26, 71)	−0.5	−1.1
June	655	98	102.9 (74, 138)	−4.9	−4.7	613	53	47.4 (27, 75)	5.6	11.9
30–39 years old										
March	720	150	143.7 (114, 176)	6.3	4.4	696	49	45.2 (30, 63)	3.8	8.5
April	720	101^b^	132.9 (103, 166)	−31.9	−24.0	694	35	39.5 (25, 57)	−4.5	−11.5
May	718	144	142.0 (108, 181)	2.0	1.4	692	50	43.5 (27, 65)	6.5	14.9
June	717	123	118.7 (88, 155)	4.3	3.6	692	48	38.3 (22, 59)	9.7	25.4
40–49 years old										
March	932	216	199.3 (163, 239)	16.7	8.4	909	75	68.6 (51, 89)	6.4	9.4
April	929	135^b^	187.1 (151, 228)	−52.1	−27.8	907	64	63.8 (46, 84)	0.2	0.2
May	927	166	198.8 (157, 247)	−32.8	−16.5	905	58	68.4 (48, 93)	−10.4	−15.2
June	926	155	178.5 (137, 226)	−23.5	−13.1	904	75	65.4 (45, 90)	9.6	14.6
50–59 years old										
March	821	197	214.7 (181, 251)	−17.7	−8.3	817	75	71.6 (54, 92)	3.4	4.8
April	822	160	188.9 (155, 225)	−28.9	−15.3	817	59	66.2 (48, 87)	−7.2	−10.8
May	823	142^b^	203.3 (164, 247)	−61.3	−30.1	819	62	69.3 (49, 93)	−7.3	−10.6
June	825	150	188.2 (150, 232)	−38.2	−20.3	820	63	61.3 (42, 85)	1.7	2.8
60–69 years old										
March	778	163	169.1 (136, 206)	−6.1	−3.6	814	73	60.7 (44, 79)	12.3	20.3
April	776	152	158.7 (125, 196)	−6.7	−4.2	812	67	58.8 (42, 78)	8.2	13.9
May	775	123^b^	185.0 (144, 232)	−62.0	−33.5	810	69	61.2 (43, 83)	7.8	12.8
June	774	136	159.5 (120, 205)	−23.5	−14.7	809	64	55.1 (37, 76)	8.9	16.1
70–79 years old										
March	744	149	154.8 (121, 192)	−5.8	−3.7	870	67	84.6 (62, 111)	−17.6	−20.8
April	744	138	150.2 (115, 190)	−12.2	−8.1	871	84	88.7 (64, 117)	−4.7	−5.3
May	746	148	164.0 (123, 212)	−16.0	−9.8	872	85	94.6 (66, 130)	−9.6	−10.2
June	747	157	151.6 (111, 200)	5.4	3.5	874	83	85.8 (58, 120)	−2.8	−3.3
Over 80 years old										
March	412	102	94.7 (70, 123)	7.3	7.7	730	63	66.1 (49, 86)	−3.1	−4.7
April	415	112	97.4 (71, 128)	14.6	15.0	732	49	62.9 (45, 83)	−13.9	−22.1
May	416	104	104.7 (74, 142)	−0.7	−0.7	733	72	63.0 (44, 86)	9.0	14.2
June	417	104	102.9 (71, 142)	1.1	1.1	734	71	66.4 (46, 91)	4.6	7.0

PI, prediction interval.

^a^95% prediction interval based on the 2.5th and 97.5th percentile of simulated distribution.

^b^Observed number deviated by 95% prediction interval.

For the total number of females, excess mortalities in March and June were positive, but those in April and May were negative; their observed numbers were within the 95% PI. For each age category in females, the highest excess mortality was 12.6 in June for under 20 years of age, with the highest relative difference of 94.3%. Excess mortality in June was positive for all age categories except for those aged 70–79 years. The lowest relative difference was −25.6% in April for the 20–29-year-old age group. However, all observed numbers in females were included in the 95% PI.

Results by prefecture are shown in eTable 1. The number of suicides during the period was below the lower bound of 95% PI in Tokyo and was observed in six prefectures, including Tokyo, in May. Excess mortality was 28 compared to 15.1 (95% PI, 7–25) in March in Yamaguchi and 84 compared to 63.1 (95% PI, 46–82) in June in Hyogo. The excess numbers from the upper bound of the PI were only 3 and 2 cases, respectively.

## DISCUSSION

In the present study, we assessed excess mortality from suicide during the early stage of the COVID-19 pandemic in Japan. Our analysis did not observe a number of suicides that exceeded the upper bound of the 95% PI, although some positive excess mortalities were observed. Although there were concerns that the spread of COVID-19 might lead to increases in suicides,^18^ our results found no significant excess mortality, but rather a downward trend in the number of suicides for both males and females during the early pandemic was noted. This decrease might have been induced by various changes, such as economic activity, communication, and social conditions under the state of emergency in Japan. For example, social isolation is thought to be associated with suicide.^19^ In Japan, people were encouraged to stay at home during this period and spend more time with their families. One possible reason is that staying at home may have had an influence on enhancing family connectedness and preventing suicide.^20^^,^^21^

Excess mortalities for the total number per month in males were below the lower bound of the 95% PI. It has been pointed out that suicide among middle-aged men in Japan was more likely to be work-related.^6^ Actually, in 2019, 58% of workers in Japan were anxious, worried, and stressed related to their work.^22^ Our results showed that negative excess mortalities were observed among the 30–69-year-old age groups amidst the early COVID-19 pandemic. One possible reason for this might be a change in their working style. For example, the percentage of telecommuting companies surged from 24.0% in March to 62.7% in April 2020.^23^ While telecommuting can increase stress,^24^^,^^25^ reducing commute times can improve the subjective well-being.^26^^,^^27^ It requires further investigations on the relationship between various style changes and stress and subjective well-being during the pandemic in Japan.

It has been pointed out that the months in which new semesters begin had a higher number of suicides among school children.^28^ The delay in the actual start of the new school year in 2020 due to school closure might have led to a negative value of excess mortalities in April for those younger than 20 years. However, those mortalities did not deviate from the PIs. This was consistent with a recent report in which no significant change was observed in suicide rates during school closure.^29^ On the other hand, we should note that there is some concern about the increase in family violence due to parental unemployment and that caused by increased time at home.^30^^,^^31^

The decrease in suicides might be temporary. Excess mortalities in June were positive and higher than those in the prior months among females. It can be inferred that the decline in suicide rates during the early pandemic months led to a gradual increase in that during later pandemics. However, the number of suicides was below the lower bound throughout the pandemic period in Tokyo. These tendencies should monitored and updated. For example, under the COVID-19 pandemic, the long-term effects of economic issues, unemployment, and business bankruptcy may exacerbate suicide.^32^

There are many factors associated with the number of suicides, including medical and psychiatric profiles,^4^ divorce rates,^33^ and monthly average temperature.^34^ Although we employed only unemployment rates with the year and month in our model, most observations were included in the PI throughout the non-pandemic period. That is, our model could provide an expected number under “normal” conditions. However, unemployment must be affected by the pandemic. The rate was 2.9% in May, which was the maximum during the pandemic period, while it was 2.4% in February 2020. To check the sensitivity of our results, we estimated ${\tilde{y}}_{t}$ as the pandemic period from the model by substituting 2.4% for the unemployment rate instead of the actual values after March 2020. We obtained consistent results in which there were no significant excess mortalities during this period as deviating from the 95% PI.

This study has several limitations. We could not identify the factors that reduced the number of suicides during the pandemic period. It is important to investigate the factors that prevent suicide in the future research. This study evaluated excess mortality only for the early pandemic period; however, the COVID-19 pandemic could affect suicide for a long time. Thus, continuous monitoring and evaluation is needed.

Despite the limitations of this study, our analysis could assess excess mortality from suicide during the early COVID-19 pandemic period in Japan. This approach could be applied not only to suicide but also for other causes of mortality to clarify the impacts of the pandemic. The findings of this study can help in the advancement of future research in preventive public health.

## APPENDIX A. SUPPLEMENTARY DATA

eTable 1. Number of suicides and excess mortality by prefecture

eFigure 1. Monthly number of suicide and 95% prediction interval by age categories for male. Dots: observed number of suicides within the 95% prediction interval; ×: observed number of suicides outside the 95% prediction interval; Solid line: predicted number of suicides in non-pandemic periods; Dashed line: predicted number of suicides in pandemic periods; Gray strip: 95% prediction interval for number of suicides based on the simulated distribution.

eFigure 2. Monthly number of suicide and 95% prediction interval by age categories for female. Dots: observed number of suicides within the 95% prediction interval; ×: observed number of suicides outside the 95% prediction interval; Solid line: predicted number of suicides in non-pandemic periods; Dashed line: predicted number of suicides in pandemic periods; Gray strip: 95% prediction interval for number of suicides based on the simulated distribution.
