# Supplementary material for: Excess Mortality From Suicide During the Early COVID-19 Pandemic Period in Japan: A Time-Series Modeling Before the Pandemic
Source: J Epidemiol. 2021 Feb 5;31(2):152–6. doi: 10.2188/jea.JE20200443 (PMC7813773; doi:10.2188/jea.JE20200443)
Supplement: Supplementary file 1 [file je-31-152-s001.pdf]

Under 20 years old

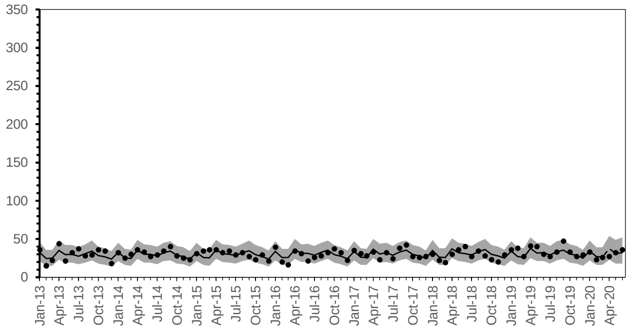

20 to 29 years old

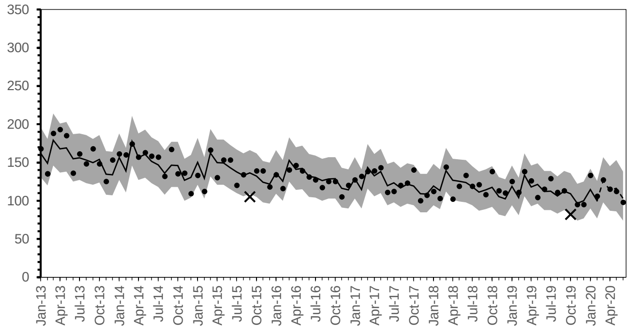

30 to 39 years old

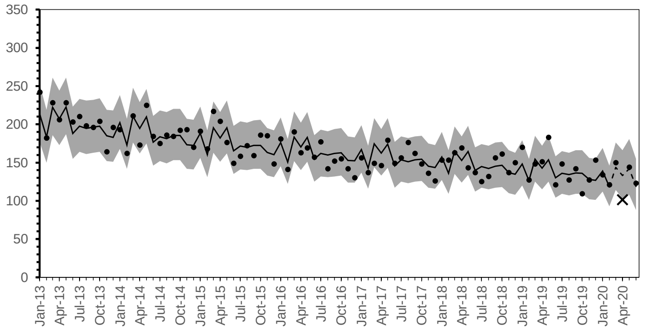

40 to 49 years old

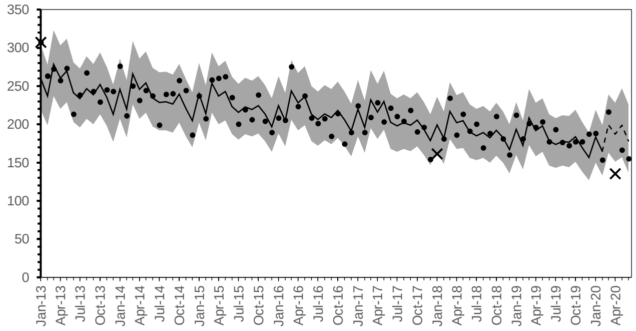

50 to 59 years old

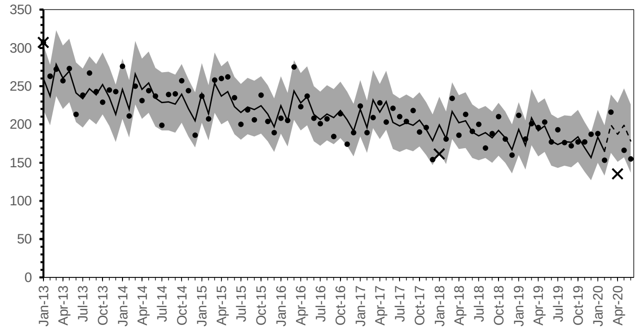

60 to 69 years old

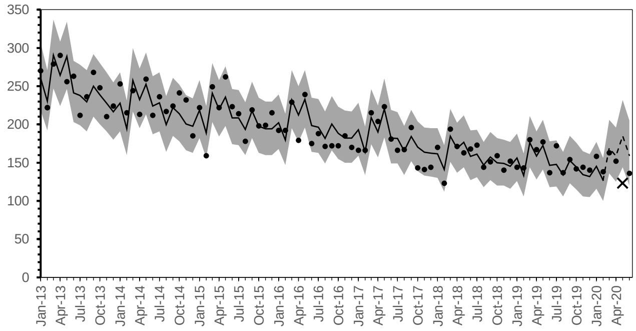

70 to 79 years old

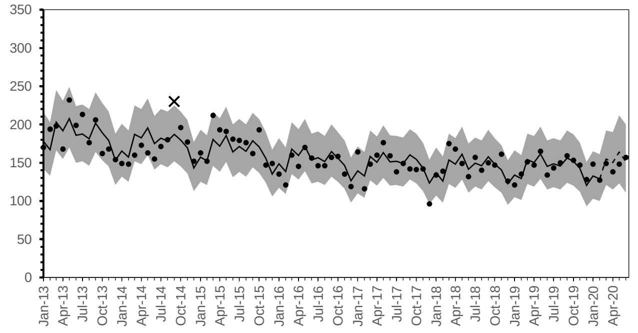

Over 80 years old

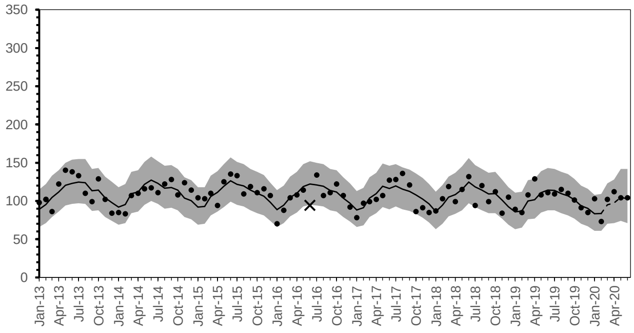

eFigure 1. Monthly number of suicide and 95% prediction interval by age categories for male. Dots: observed number of suicides within the 95% prediction interval; ×: observed number of suicides outside the 95% prediction interval; Solid line: predicted number of suicides in non-pandemic periods; Dashed line: predicted number of suicides in pandemic periods; Gray strip: 95% prediction interval for number of suicides based on the simulated distribution.

Under 20 years old

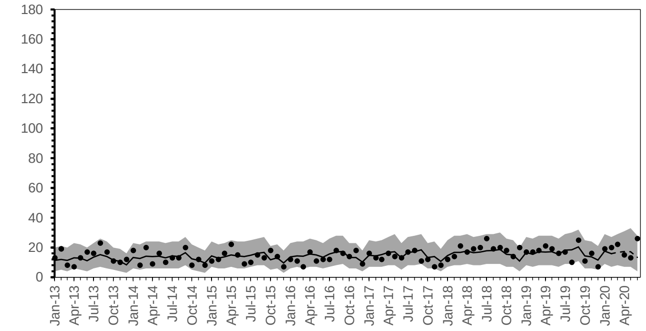

20 to 29 years old

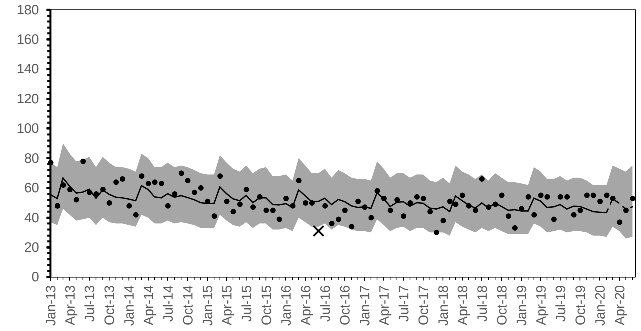

30 to 39 years old

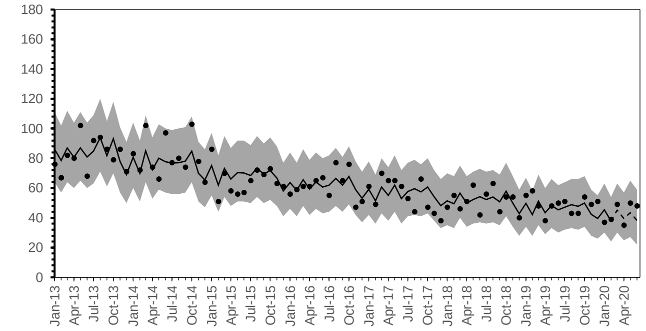

40 to 49 years old

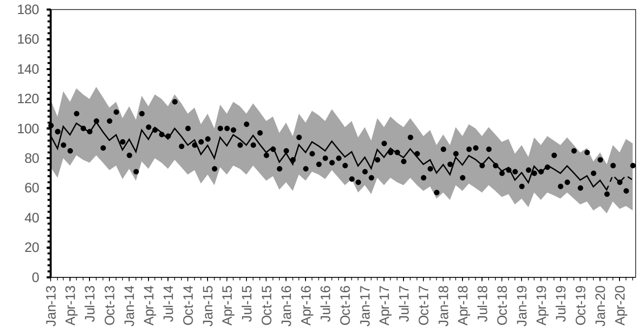

50 to 59 years old

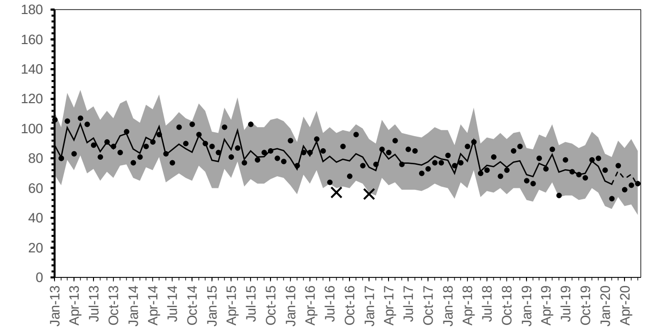

60 to 69 years old

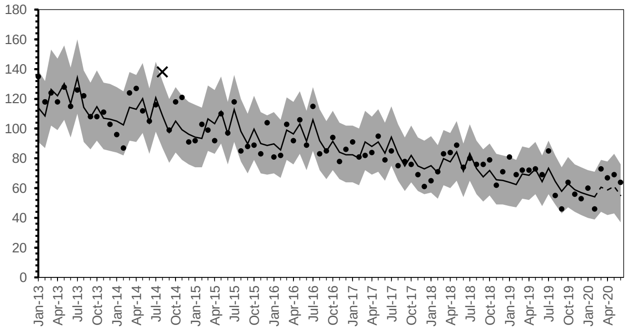

70 to 79 years old

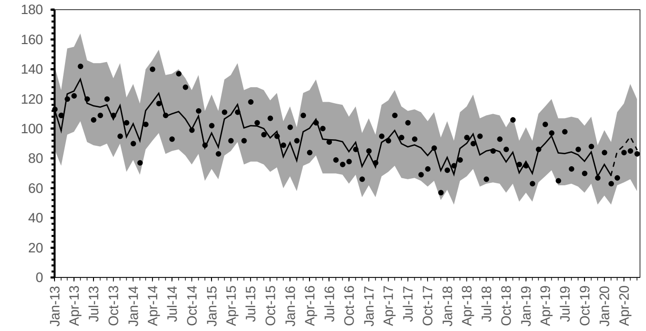

Over 80 years old

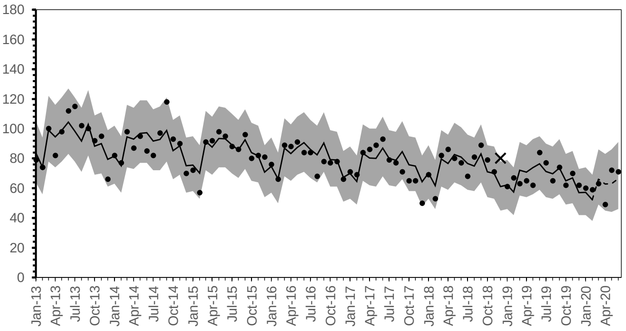

eFigure 2. Monthly number of suicide and 95% prediction interval by age categories for female. Dots: observed number of suicides within the 95% prediction interval; ×: observed number of suicides outside the 95% prediction interval; Solid line: predicted number of suicides in non-pandemic periods; Dashed line: predicted number of suicides in pandemic periods; Gray strip: 95% prediction interval for number of suicides based on the simulated distribution.

eTable 1. Number of suicides and excess mortality by prefecture

Prefecture	March				April				May				June			
	Observed number	Prediction number (95% PI)[a]	Excess mortality	Relative difference (%)	Observed number	Prediction number (95% PI)[a]	Excess mortality	Relative difference (%)	Observed number	Prediction number (95% PI)[a]	Excess mortality	Relative difference (%)	Observed number	Prediction number (95% PI)[a]	Excess mortality	Relative difference (%)
Hokkaido	69	70.7 (53, 91)	-1.7	-2.4	69	67.2 (50, 86)	1.8	2.7	63	74.3 (53, 99)	-11.3	-15.2	69	66.7 (49, 86)	2.3	3.4
Aomori-ken	22	18.9 (10, 30)	3.1	16.4	20	18.8 (10, 29)	1.2	6.4	18	15.4 (7, 27)	2.6	16.9	13	17.8 (9, 28)	-4.8	-26.9
Iwate-ken	27	21.3 (12, 33)	5.7	26.8	21	19.0 (10, 29)	2.0	10.5	13	22.9 (12, 37)	-9.9	-43.3	19	18.8 (10, 29)	0.2	1.1
Miyagi-ken	44	31.6 (19, 47)	12.4	39.2	29	29.3 (17, 44)	-0.3	-1.0	30	36.8 (21, 57)	-6.8	-18.5	30	28.1 (16, 42)	1.9	6.8
Akita-ken	16	14.8 (7, 25)	1.2	8.1	13	14.6 (7, 24)	-1.6	-10.9	15	13.5 (6, 24)	1.5	11.1	11	13.9 (7, 23)	-2.9	-20.8
Yamagata-ken	11	16.3 (7, 28)	-5.3	-32.6	14	15.9 (7, 27)	-1.9	-11.9	13	17.5 (7, 33)	-4.5	-25.7	19	13.3 (5, 23)	5.7	42.7
Fukushima-ken	33	27.7 (15, 43)	5.3	19.1	17	25.3 (14, 40)	-8.3	-32.8	38	28.3 (15, 47)	9.7	34.3	18	27.0 (15, 42)	-9.0	-33.3
Ibaraki-ken	40	36.5 (23, 53)	3.5	9.6	19[b]	39.0 (25, 55)	-20.0	-51.2	28	32.8 (19, 51)	-4.8	-14.6	29	38.3 (24, 54)	-9.3	-24.3
Tochigi-ken	28	28.0 (16, 42)	0.0	0.0	22	29.9 (18, 44)	-7.9	-26.4	16[b]	31.3 (18, 49)	-15.3	-48.9	22	24.4 (14, 37)	-2.4	-9.9
Gumma-ken	22	31.7 (20, 46)	-9.7	-30.6	38	30.1 (19, 43)	7.9	26.3	27	31.8 (19, 49)	-4.8	-15.1	26	24.6 (14, 37)	1.4	5.7
Saitama-ken	89	89.7 (67, 115)	-0.7	-0.8	80	83.9 (62, 108)	-3.9	-4.6	61[b]	91.3 (66, 121)	-30.3	-33.2	83	76.2 (56, 99)	6.8	8.9
Chiba-ken	92	73.0 (53, 96)	19.0	26.0	73	70.9 (51, 93)	2.1	3.0	74	67.6 (46, 93)	6.4	9.5	73	63.9 (46, 85)	9.1	14.2
Tokyo-to	85[b]	153.3 (107, 208)	-68.3	-44.6	24[b]	146.8 (102, 198)	-122.8	-83.7	71[b]	141.5 (93, 202)	-70.5	-49.8	43[b]	153.9 (108, 207)	-110.9	-72.1
Kanagawa-ken	90	81.9 (60, 106)	8.1	9.9	78	84.0 (62, 107)	-6.0	-7.1	82	77.1 (54, 104)	4.9	6.4	83	87.6 (66, 112)	-4.6	-5.2
Niigata-ken	29	37.8 (24, 54)	-8.8	-23.3	29	34.3 (22, 49)	-5.3	-15.5	33	34.9 (21, 53)	-1.9	-5.4	33	36.1 (23, 51)	-3.1	-8.6
Toyama-ken	19	14.1 (6, 25)	4.9	34.7	18	13.7 (6, 24)	4.3	31.4	21	12.4 (4, 24)	8.6	69.6	19	14.6 (6, 25)	4.4	30.2
Ishikawa-ken	15	17.4 (8, 29)	-2.4	-13.8	11	14.0 (6, 24)	-3.0	-21.4	8	17.8 (8, 33)	-9.8	-55.1	15	14.1 (6, 24)	0.9	6.4
Fukui-ken	16	8.8 (3, 17)	7.2	81.9	10	9.4 (3, 17)	0.6	6.4	4	7.4 (2, 16)	-3.4	-46.2	12	10.2 (4, 19)	1.8	17.6
Yamanashi-ken	10	13.2 (5, 24)	-3.2	-24.3	5	10.9 (4, 20)	-5.9	-54.0	3[b]	11.2 (4, 23)	-8.2	-73.2	6	10.6 (4, 20)	-4.6	-43.3
Nagano-ken	23	32.0 (20, 46)	-9.0	-28.1	16	26.9 (16, 40)	-10.9	-40.6	38	31.2 (18, 48)	6.8	21.8	18	22.9 (13, 34)	-4.9	-21.4
Gifu-ken	27	25.2 (15, 38)	1.8	7.1	15	24.2 (14, 36)	-9.2	-38.0	22	26.1 (15, 41)	-4.1	-15.7	17	21.0 (12, 32)	-4.0	-19.1
Shizuoka-ken	49	52.5 (37, 70)	-3.5	-6.7	46	45.0 (31, 61)	1.0	2.2	55	57.8 (39, 81)	-2.8	-4.8	34	47.9 (33, 65)	-13.9	-29.0
Aichi-ken	91	95.6 (73, 121)	-4.6	-4.8	79	92.6 (71, 117)	-13.6	-14.7	77[b]	110.6 (82, 143)	-33.6	-30.4	72	89.8 (69, 114)	-17.8	-19.8
Mie-ken	27	23.7 (12, 37)	3.3	13.9	20	23.0 (12, 36)	-3.0	-13.0	20	25.0 (12, 43)	-5.0	-20.0	25	25.1 (14, 40)	-0.1	-0.4
Shiga-ken	14	20.8 (11, 33)	-6.8	-32.7	20	16.1 (8, 25)	3.9	24.2	10	21.1 (10, 36)	-11.1	-52.7	17	17.5 (9, 28)	-0.5	-2.9
Kyoto-fu	27	22.3 (13, 34)	4.7	21.1	26	21.5 (12, 32)	4.5	20.9	20	21.6 (12, 34)	-1.6	-7.4	20	24.7 (15, 36)	-4.7	-19.0
Osaka-fu	85	77.9 (54, 106)	7.1	9.1	71	70.5 (49, 96)	0.5	0.7	100	78.8 (53, 112)	21.2	26.9	86	77.6 (54, 106)	8.4	10.8
Hyogo-ken	59	77.7 (58, 100)	-18.7	-24.1	61	68.8 (50, 89)	-7.8	-11.3	67	79.5 (57, 106)	-12.5	-15.7	84[b]	63.1 (46, 82)	20.9	33.1
Nara-ken	11	15.0 (7, 25)	-4.0	-26.7	10	14.0 (6, 24)	-4.0	-28.6	13	13.0 (5, 25)	0.0	0.0	10	13.7 (6, 24)	-3.7	-27.0
Wakayama-ken	13	11.1 (5, 19)	1.9	17.1	12	14.6 (7, 24)	-2.6	-17.8	11	11.5 (4, 22)	-0.5	-4.4	14	14.1 (7, 24)	-0.1	-0.7
Tottori-ken	3	5.7 (1, 13)	-2.7	-47.5	4	6.6 (2, 14)	-2.6	-39.1	2	6.0 (1, 15)	-4.0	-66.4	3	4.0 (0, 10)	-1.0	-25.1
Shimane-ken	6	8.0 (3, 15)	-2.0	-24.8	6	7.2 (2, 14)	-1.2	-16.7	9	7.3 (2, 16)	1.7	23.2	11	9.2 (3, 17)	1.8	19.5
Okayama-ken	24	21.8 (12, 34)	2.2	10.1	18	20.2 (11, 31)	-2.2	-10.9	14	19.9 (10, 33)	-5.9	-29.6	17	22.6 (13, 34)	-5.6	-24.8
Hiroshima-ken	37	32.4 (20, 47)	4.6	14.2	25	33.3 (21, 47)	-8.3	-24.9	23	33.9 (20, 51)	-10.9	-32.2	28	36.5 (23, 52)	-8.5	-23.3
Yamaguchi-ken	28[b]	15.1 (7, 25)	12.9	85.4	18	13.0 (6, 22)	5.0	38.3	18	13.6 (6, 25)	4.4	32.5	15	18.1 (9, 29)	-3.1	-17.2
Tokushima-ken	9	8.2 (3, 16)	0.8	9.8	6	7.3 (2, 14)	-1.3	-17.7	4	7.9 (2, 17)	-3.9	-49.1	8	6.6 (2, 13)	1.4	21.4
Kagawa-ken	9	12.0 (5, 21)	-3.0	-25.0	13	14.3 (7, 24)	-1.3	-9.1	8	11.7 (4, 22)	-3.7	-31.6	8	14.3 (7, 24)	-6.3	-44.0
Ehime-ken	16	16.6 (8, 27)	-0.6	-3.6	18	16.0 (8, 26)	2.0	12.5	18	13.6 (6, 24)	4.4	32.4	12	17.8 (9, 28)	-5.8	-32.7
Kochi-ken	8	8.0 (2, 17)	0.0	0.0	8	8.6 (2, 18)	-0.6	-6.9	6	9.6 (2, 22)	-3.6	-37.5	10	12.7 (5, 24)	-2.7	-21.3

Fukuoka-ken	59	66.6 (48, 88)	-7.6	-11.4	57	57.2 (40, 77)	-0.2	-0.3	63	61.0 (41, 84)	2.0	3.3	62	53.3 (37, 72)	8.7	16.3
Saga-ken	11	13.1 (6, 23)	-2.1	-16.0	6	12.2 (5, 22)	-6.2	-50.7	6	14.0 (5, 27)	-8.0	-57.0	6	11.5 (5, 20)	-5.5	-48.0
Nagasaki-ken	11	9.8 (4, 18)	1.2	12.3	15	10.4 (4, 18)	4.6	44.4	9	8.1 (2, 16)	0.9	11.2	9	11.0 (4, 19)	-2.0	-18.2
Kumamoto-ken	32	27.4 (16, 42)	4.6	16.8	22	25.9 (15, 39)	-3.9	-15.1	18	27.3 (14, 45)	-9.3	-34.1	14	18.3 (9, 29)	-4.3	-23.6
Oita-ken	13	20.3 (11, 32)	-7.3	-36.0	18	15.4 (8, 25)	2.6	16.9	7[b]	21.7 (11, 37)	-14.7	-67.8	19	14.5 (7, 24)	4.5	30.9
Miyazaki-ken	16	19.0 (10, 30)	-3.0	-15.8	11	15.1 (7, 25)	-4.1	-27.1	22	17.7 (8, 31)	4.3	24.3	12	11.6 (5, 20)	0.4	3.4
Kagoshima-ken	23	22.4 (12, 35)	0.6	2.7	23	22.7 (13, 36)	0.3	1.3	28	25.7 (13, 43)	2.3	8.9	26	24.3 (14, 38)	1.7	7.0
Okinawa-ken	14	14.8 (6, 26)	-0.8	-5.4	13	18.7 (9, 31)	-5.7	-30.5	13	16.1 (7, 30)	-3.1	-19.3	16	12.9 (5, 23)	3.1	24.1

PI, prediction interval.

[a]95% prediction interval based on the 2.5th and 97.5th percentile of simulated distribution.

[b]Observed number deviated by 95% prediction interval.
